# Association of the Angiotensin-Converting Enzyme Insertion/Deletion Polymorphism with Increased Susceptibility to Vasculitis: A Systematic Review and Meta-Analysis

**DOI:** 10.31138/mjr.111125.isv

**Published:** 2026-06-10

**Authors:** Praveen Kumar Chandra Sekar, Ramakrishnan Veerabathiran

**Affiliations:** Human Cytogenetics and Genomics Laboratory, Faculty of Allied Health Sciences, Chettinad Hospital and Research Institute, Chettinad Academy of Research and Education, Kelambakkam, Tamil Nadu, India

**Keywords:** vasculitis, Henoch–Schönlein purpura nephritis, Behçet’s disease, Henoch-Schönlein purpura, meta-analysis

## Abstract

**Objective:**

This meta-analysis aimed to evaluate the association between the angiotensin-converting enzyme (ACE) insertion/deletion (I/D) polymorphism and susceptibility to vasculitis, including Henoch– Schönlein purpura (HSP), Henoch–Schönlein purpura nephritis (HSPN), and Behçet’s disease (BD).

**Methods:**

Relevant studies were systematically retrieved from PubMed, Embase, and Google Scholar until November 2025. Odds ratios (ORs) and 95% confidence intervals (CIs) were calculated using different genetic models. Heterogeneity was assessed using the Cochrane Q test and I^2^ statistics, and random-effects models were applied. Statistical analyses were performed using the Review Manager (version 5.4).

**Results:**

Fifteen eligible studies, including five on HSP, four on HSPN, and six on BD, were included, with a total of 1,536 cases and 1,947 controls. Pooled analysis demonstrated a significant association between the ACE I/D polymorphism and an increased risk of vasculitis (D vs. I, OR = 1.42, 95% CI 1.15–1.76), with stronger associations observed in Caucasian populations. Subgroup analysis revealed that the D allele was significantly associated with higher susceptibility to HSP and BD, whereas a modest association was detected for HSPN in the allelic comparison model.

**Conclusion:**

The findings suggest that the ACE D allele may be a genetic risk factor for vasculitic disorders, especially HSP and BD, supporting the possible involvement of the renin–angiotensin system in the development of autoimmune vascular inflammation.

## INTRODUCTION

Vasculitis is a diverse collection of disorders characterised by inflammation of the blood vessel walls, resulting in vascular damage and impaired organ perfusion.^1^ Vasculitis affects approximately 20–50 individuals per million annually, with specific subtypes exhibiting variable prevalence rates worldwide. ^2^ For instance, Henoch–Schönlein purpura (HSP) is the most prevalent form of vasculitis in the paediatric population, with an annual incidence of 10–20 cases per 100,000 children. Giant cell arteritis is the most common form of vasculitis in adults. Behçet’s disease (BD) is more prevalent in regions along the ancient Silk Road, affecting approximately 1 in 1,000 individuals, and is less common in Western countries than in Asia.^4^ Henoch-Schönlein purpura nephritis (HSPN), a severe renal complication of HSP, occurs in approximately 20–50% of patients diagnosed with HSP.^5^ The aetiology of vasculitis is multifactorial and involves genetic, environmental, and immunological factors.^6^ Polymorphisms in genes regulating the renin-angiotensin system (RAS) have been correlated with the risk of vasculitis.^7^ The angiotensin-converting enzyme (*ACE*) gene is located on chromosome 17q23 and encodes an essential enzyme in RAS. This enzyme facilitates the conversion of angiotensin I to angiotensin II and degrades bradykinin via the kallikrein-kinin system.^8^ Angiotensin II, the main effector of the RAS, is involved in the onset and advancement of renal disorders.^9^ Moreover, it functions as a potent pro-inflammatory mediator that amplifies and sustains immune responses. A widely studied genetic variation in the *ACE* gene is the insertion/deletion (I/D) polymorphism, which is categorised by either the presence (insertion, I) or absence (deletion, D) of a 287-base pair Alu repeat sequence within intron 16.^10^ Individuals carrying the DD genotype exhibit approximately two-fold higher ACE levels in plasma and tissues than those with the II genotype, with ID heterozygotes showing intermediate levels.^11^ This increased ACE activity leads to enhanced conversion of angiotensin I to Angiotensin II and accelerated degradation of bradykinin, resulting in elevated angiotensin II bioavailability.^12^ Angiotensin II is a potent vasoactive and pro-inflammatory mediator that promotes endothelial dysfunction, oxidative stress, vascular remodelling, and inflammatory cytokine release, thereby contributing to vascular inflammation and injury.^13^ Although no specific circulating ACE threshold has been established as a diagnostic marker of endothelial damage, elevated ACE activity, particularly in individuals carrying the DD genotype, has been consistently associated with impaired nitric oxide bioavailability, increased oxidative stress, and vascular inflammation. Thus, ACE activity is best regarded as a risk-modifying or supportive biomarker reflecting susceptibility to vascular injury, rather than a standalone biomarker of inflammation. Given these biological effects, this polymorphism may affect susceptibility to various inflammatory diseases, including vasculitis. The potential mechanisms linking the D allele to vasculitis involve excessive angiotensin II production, which promotes vascular remodelling, pro-inflammatory cytokine release, increased oxidative stress, and key factors in vascular inflammation and immune dysregulation.^14^ These mechanisms are particularly relevant to small- and medium-vessel vasculitides such as HSP, HSPN, and BD, in which endothelial dysfunction, immune complex deposition, and vascular inflammation play central roles in disease pathogenesis. In contrast, ANCA-associated vasculitides are primarily driven by autoantibody-mediated neutrophil activation and necrotising vascular inflammation, mechanisms that may be less directly influenced by RAS dysregulation.^15^ This pathophysiological distinction may partly explain why associations between ACE I/D polymorphism and disease susceptibility are more consistently reported in HSP, HSPN, and BD than in ANCA-associated vasculitides.

Numerous studies have investigated the correlation between this polymorphism and susceptibility to vasculitis, but the results have been inconclusive.^16,17^ While some studies have suggested a substantial association between the increased risk of developing vasculitis and the D allele, others have reported no such association. These discrepancies may be due to variations in the study populations, sample sizes, ethnic backgrounds, and methodological variations that influence disease susceptibility and progression. Notably, HSP, HSPN, and BD have been examined for this polymorphism, although the findings are inconclusive. Therefore, a comprehensive systematic meta-analysis is essential to synthesise the existing data and clarify the potential role of this polymorphism in the pathogenesis of vasculitis. The current meta-analysis aimed to evaluate the association between the ACE I/D polymorphism and susceptibility to immune-mediated vasculitis. The scope of this review was limited to the most frequently studied forms of vasculitis in genetic association studies, namely HSP, HSPN, and BD. Given the rarity of vasculitic disorders and the limited availability of genetic studies, subgroup analyses were performed according to disease type and ethnicity, where sufficient data were available. Understanding the genetic contribution to vasculitis susceptibility may provide insights into the disease mechanisms and inform future genetic and therapeutic research in this field.

## MATERIALS AND METHODS

This systematic review and meta-analysis were conducted and reported in accordance with the Preferred Reporting Items for Systematic Reviews and Meta-Analyses (PRISMA) 2021 guidelines, ensuring transparency, reproducibility, and comprehensive reporting, in line with the EQUATOR Network recommendations. The review protocol was prospectively registered in the International Prospective Register of Systematic Reviews (PROSPERO) under registration number CRD420251027427, which details the review objectives, eligibility criteria, methodological approach, and planned analyses (https://www.crd.york.ac.uk/PROS-PERO/view/CRD420251027427).

### Ethical consideration

Ethical approval was not required for this study as it relied solely on previously published case-control studies. As this was a meta-analysis, the requirement for patient consent was waived.

### Search strategy and study selection

Three primary electronic databases, Medline/PubMed, Embase, Scopus, Web of Science, and the Directory of Open Access Journals (DOAJ), were systematically searched to identify relevant studies investigating the association between genetic variants and vasculitis. The literature search was initially conducted and subsequently updated to include studies published until November 2025. The search strategy was developed according to the established recommendations for comprehensive systematic reviews. Searches were conducted using various combinations of Medical Subject Headings (MeSH) terms and free-text keywords, including “vasculitis,” “Henoch–Schönlein purpura,” “Henoch–Schönlein purpura nephritis,” “Behçet’s disease,” “angiotensin-converting enzyme,” “ACE,” “insertion/deletion,” “polymorphism,” “SNP,” “HSP,” “HSPN,” “BD,” and “case–control association.” Boolean operators (“AND,” “OR”) were applied to optimise the search sensitivity and specificity. To ensure completeness, the reference lists of all eligible articles and relevant reviews were manually screened to identify additional studies that were not captured through the database searches.

### Eligibility criteria

Studies were considered eligible for inclusion in the meta-analysis if they met the following criteria.

1. Study design: Case–control studies evaluating genetic polymorphisms in patients with vasculitis and healthy controls.
2. Participants: Individuals diagnosed with vasculitis based on clinical evidence of systemic inflammation and vascular involvement were included.
3. Age group: Both paediatric and adult populations were eligible for inclusion in the study. Studies on HSP and HSPN predominantly involve children, whereas studies on Behçet’s disease mainly include adult patients. No age-based restrictions were imposed.
4. Genotyping methods: Studies in which vasculitis diagnosis was established using accepted clinical or histopathological criteria, ACE I/D genotyping was performed using PCR-based molecular techniques, including PCR-RFLP or related PCR-based methods
5. Language: Articles published in English.

### Exclusion criteria

Studies were excluded if they met any of the following criteria:

Non–case–control study designs, including reviews, editorials, meta-analyses, and case reports.

Duplicate publications or overlapping datasets. Studies of insufficient methodological quality based on predefined quality assessment criteria.

Studies lacking essential genotype or allele frequency data required for meta-analysis.

Studies with inadequate control for potential confounding factors.

Non-human studies, including animal or in vitro experiments, unless directly relevant to the research question.

#### Data extraction

The studies included in our analysis documented the authors’ names, country of origin, year of publication, study design, sample size, and genotyping method. PKCS independently performed the data extraction, and any inconsistencies were resolved through mutual discussion. We assessed the quality of the included studies using NOS.^18^

### Quality assessment

Hardy–Weinberg equilibrium (HWE) was assessed in the control groups of the included studies using p-value analysis to evaluate the consistency of the genotype distributions. The methodological quality of the eligible observational case–control studies was assessed using the Newcastle–Ottawa Scale (NOS), which evaluates study selection, comparability of cases and controls, and ascertainment of exposure. Studies with NOS scores ≥6 were considered to have moderate-to-high methodological quality. Potential sources of bias were systematically evaluated, as methodological rigor is critical for the reliability of pooled effect estimates in meta-analyses. The results of the NOS-based quality assessment were summarised and graphically presented to facilitate comparisons across studies. All quality assessments were conducted in accordance with the reporting and appraisal principles recommended by the EQUATOR Network.

### Statistical analysis

This meta-analysis aimed to evaluate the association between the ACE I/D polymorphism and susceptibility to vasculitis. Odds ratios (ORs) with corresponding 95% CIs were calculated to estimate the strength of associations, and a two-sided P value < 0.05 was considered statistically significant. For all genetic analyses, the I allele and II genotype were used as reference categories, and the D allele was considered the risk allele. Between-study heterogeneity was assessed using Cochran’s Q test and quantified using the I^2^ statistic, with I^2^ values of <25%, 25–50%, and >50% indicating low, moderate, and substantial heterogeneity, respectively. A fixed-effects model was applied when heterogeneity was low, whereas a random-effects model was used in the presence of significant heterogeneity (I^2^ > 50% or P < 0.10 for the Q-test). Publication bias was evaluated using funnel plot asymmetry and Egger’s regression test, with a P-value < 0.05 indicating potential small-study effects. Sensitivity analyses were conducted to assess the stability of pooled estimates. All statistical analyses were performed using Review Manager (RevMan) version 5.4 and G*Power version 3.1.

### Power Analysis

Power analysis was performed on the compiled data using a 95% CI and significance level (α) of 0.05. Statistical power was calculated based on the sample sizes of the case and control groups for each selected gene, which were assessed collectively and individually. We employed G*Power 3.1 owing to its reliability and widespread acceptance in power analyses of various statistical tests. Additionally, the online tool available at http://www.power-analysis.com/ was used to cross-verify the accuracy of our calculations. This comprehensive approach enabled us to finalise the minimum sample size necessary to achieve adequate statistical power, thereby minimising the potential for Type II errors and strengthening the validity and reliability of our results.

### Gene co-expression network

STRING (https://string-db.org/) is an online database that enables the construction of gene co-expression and protein-protein interaction networks by integrating high-throughput experimental data with computational prediction models.^19^ In this study, we used STRING to construct a gene co-expression network for ACE, applying a moderate confidence threshold of 0.400 as the minimum required interaction score.

## RESULTS

**Figure 1.**
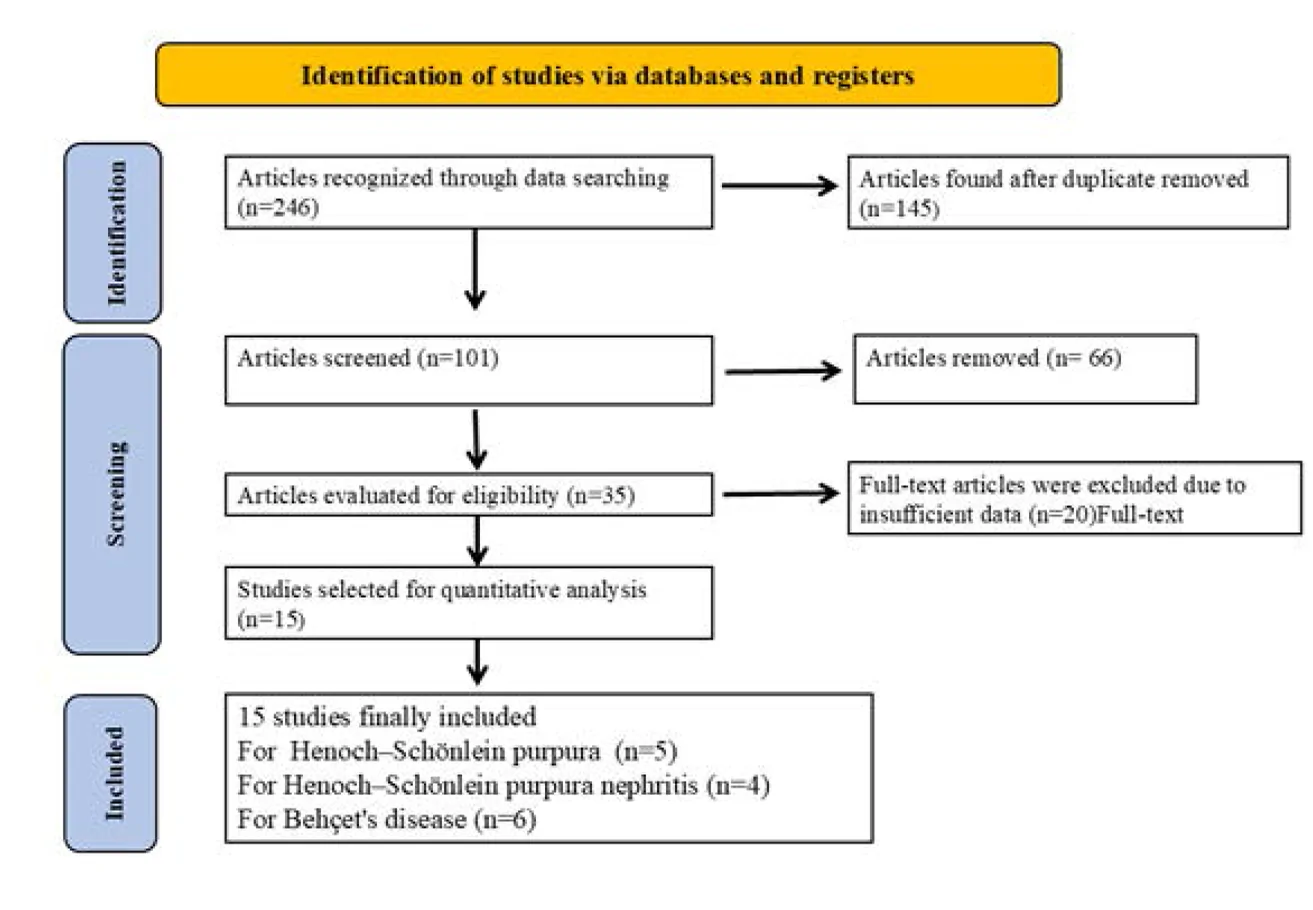
Flow chart of literature screening investigating ACE I/D gene polymorphism with vasculitis. (above)

We identified 246 citations in total. After reviewing the abstracts and titles, 145 articles were excluded. A total of 101 complete manuscripts were thoroughly examined, of which 86 were excluded for the following reasons: 35 were review articles, 13 had unrelated study objectives, 21 focused solely on protein expression levels, and 20 lacked genotyping data (**Figure 1**). In total, 15 eligible studies were selected and incorporated into the comprehensive analysis (**Table 1**). Specifically, five studies investigated gene polymorphisms related to HSP, four examined the I/D polymorphisms of the ACE gene in relation to HSPN, and five assessed gene polymorphisms associated with BD.

**Table 1.** Characteristics of the studies for the association of ACE I/D gene polymorphism with vasculitis risk.

First author	Year	Country/Ethnicity	Genotyping Method	Case /control	Genotype frequency						Allele frequency				HWE
					Case			Control			Case		Control		
*ACE* I/D polymorphism with HSP															
					II	ID	DD	II	ID	DD	I	D	I	D	
Liu et al.	2010	China/Asian	RFLP-PCR	146/217	43	77	26	94	102	21	163	129	290	144	0.37
Mohammadian et al.	2017	Iran/Asian	RFLP-PCR	47/74	14	16	17	16	28	30	44	50	60	88	0.06
Nalbantoglu et al.	2013	Turkey/Caucasian	PCR-RFLP	139/72	20	58	61	20	36	16	98	180	76	68	0.97
Ozkaya et al.	2004	USA/Caucasian	PCR	114/164	8	88	18	28	117	19	104	124	173	155	0.847
Taskin et al.	2024	Turkey/Caucasian	PCR-RFLP	53/50	8	22	23	8	24	18	38	68	40	60	1
*ACE* I/D polymorphism with HSPN					II	ID	DD	II	ID	DD	I	D	I	D	
Di et al.	2012	China/Asian	RFLP-PCR	160/137	37	112	11	25	73	39	186	134	123	151	0.81
Liu et al.	2010	China/Asian	PCR-RFLP	61/217	17	33	11	94	102	21	67	55	290	144	0.78
Ozkaya et al.	2004	USA/Caucasian	PCR-RFLP	44/164	7	29	8	28	117	19	43	45	173	155	30.4
Zhou et al.	2004	China/Asian	PCR-RFLP	103/100	41	32	30	55	39	6	114	92	149	51	0.06
*ACE* I/D polymorphism with BD					II	ID	DD	II	ID	DD	I	D	I	D	
Chang et al.	2004	South Korea/Asian	PCR-RFLP	70/106	25	28	17	42	44	20	78	62	128	84	1.85
Dursun et al.	2009	Turkey/Caucasian	PCR-RFLP	73/90	12	29	32	23	35	32	53	93	81	99	4.13
Ozturk et al.	2004	Turkey/Caucasian	PCR-RFLP	90/30	12	56	22	5	16	9	80	100	26	34	0.22
Rouhi et al.	2019	Iran/Asian	PCR-RFLP	135/76	24	55	56	17	29	30	103	167	63	89	3.47
Turgut et al.	2005	Turkey/Caucasian	PCR-RFLP	35/150	2	7	26	19	44	87	11	59	82	218	10.2
Yigit et al.	2013	Turkey/Caucasian	PCR-RFLP	266/300	112	50	104	128	95	77	274	258	351	249	36.2

### Risk of bias

The methodological quality of the included observational case–control studies was assessed using NOS, as summarised in **Supplementary Table S1**. Each study was evaluated across three domains: selection of study groups, comparability of cases and controls, and exposure ascertainment. Overall, most studies demonstrated moderate to high methodological quality, indicating a low likelihood of major systematic bias. Variability in quality scores primarily reflects differences in case definition, control selection, and adjustment for potential confounders, which are common challenges in retrospective genetic association studies. Importantly, genotyping methods were consistently well described and based on validated molecular techniques, supporting the reliability of the exposure assessment. Collectively, the NOS-based evaluation suggests that the included studies were methodologically sound and suitable for inclusion in the pooled meta-analysis.

### ACE I/D polymorphism with HSP

**Figure 2.**
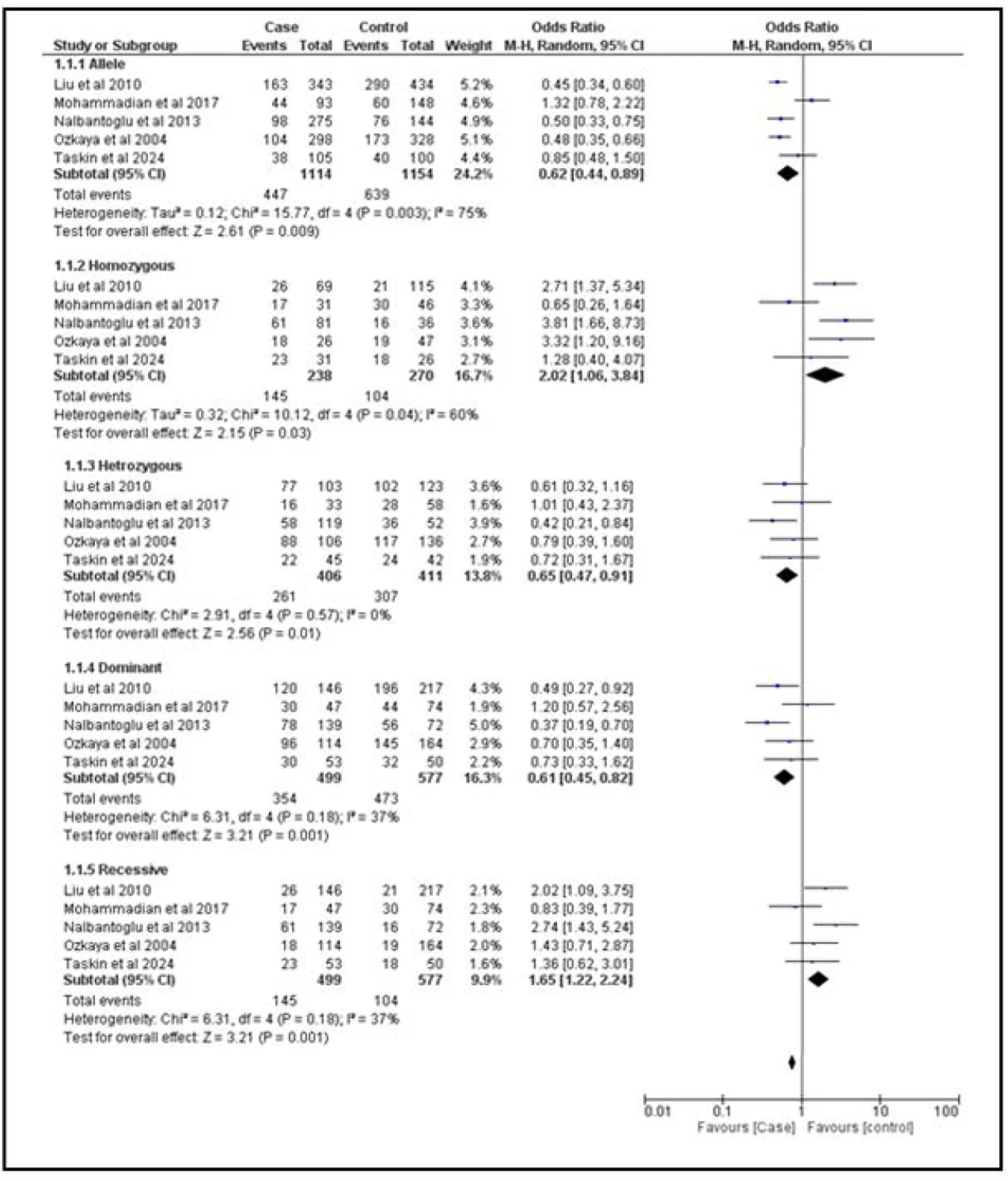
Forest plot showing an association between the ACE I/D gene polymorphism and Henoch–Schönlein purpura risk in the genetic models. (below)

This polymorphism has been extensively studied in HSP. A meta-analysis of five studies, which included 499 HSP cases and 577 controls,^20–24^ showed a substantial association with the allele (P = 0.009, OR = 0.62, 95% CI [0.44, 0.89], I^2^ = 75%). A significant association was found in the homozygote group (P = 0.03, OR = 2.02, 95% CI [1.06, 3.84], I^2^ = 60%) using random-effects models. Similar results were observed in the recessive (P = 0.001, OR = 1.65, 95% CI [1.22, 2.24], I^2^ = 37%), dominant (P = 0.001, OR = 0.61, 95% CI [0.45, 0.82], I^2^ = 37%), and heterozygote groups (P = 0.01, OR = 0.87, 95% CI [0.43, 1.76], I^2^ = 81%) using fixed-effects models (**Figure 2**). Subgroup analyses of the Asian and Caucasian populations indicated that the allelic model for Caucasian populations demonstrated a substantial association (P = 0.0006, OR = 0.66, 95% CI [0.52–0.83], I^2^ = 40%). In contrast, the Asian population demonstrated no significant correlation (P = 0.7, OR = 0.87, 95% CI [0.43, 1.76], I^2^ = 81%) (**Table 2**).

**Table 2.** Subgroup analysis for ACE I/D gene polymorphism with vasculitis risk.

Polymorphism	Genetic Model	Ethnicity	Test of association			Test of heterogeneity			I^2^	Publication bias P (Egger's test)
			Number of Studies	OR	95 % CI	P	Model	P		
*ACE* I/D with HSP	Allele contrast (I vs. D)	Overall	5	0.7285	[0.5502; 0.9646]	0.027012	Random	0.0528	0.5725	0.3582
		Asian	2	0.8714	[0.4310; 1.7618]	0.701452	Random	0.0193	0.8173	0
		Caucasian	3	0.6626	[0.5228; 0.8397]	0.000661	Fixed	0.1857	0.4061	0.9261
	Recessive model (II vs. ID+DD)	Overall	5	0.624	[0.3938; 0.9888]	0.044673	Random	0.0967	0.4913	0.5541
		Asian	2	0.8609	[0.3145; 2.3562]	0.770603	Random	0.0321	0.7823	0
		Caucasian	3	0.4798	[0.2976; 0.7734]	0.002574	Fixed	0.373	0.9273	0.4098
*ACE* I/D with BD	Dominant model (II+ID vs. DD)	Overall	6	0.6641	[0.5243; 0.8411]	0.000688	Fixed	0.3624	0.0842	0.2291
		Asian	2	0.8403	[0.5351; 1.3196]	0.449866	Fixed	0.6155	0	0
		Caucasian	4	0.6075	[0.4603; 0.8018]	0.000431	Fixed	0.2877	0.2037	0.3827
	Recessive model (II+ID vs. DD)	Overall	6	0.8471	[0.6620; 1.0840]	0.187288	Fixed	0.7524	0.5925	0.0243
		Asian	2	0.8023	[0.5038; 1.2776]	0.353375	Fixed	0.8007	0.5625	0
		Caucasian	4	0.8653	[0.6469; 1.1574]	0.329584	Fixed	0.4713	0.8173	0.1294

Reference allele: I; Reference genotype: II

### ACE I/D polymorphism with HSPN

**Figure 3.**
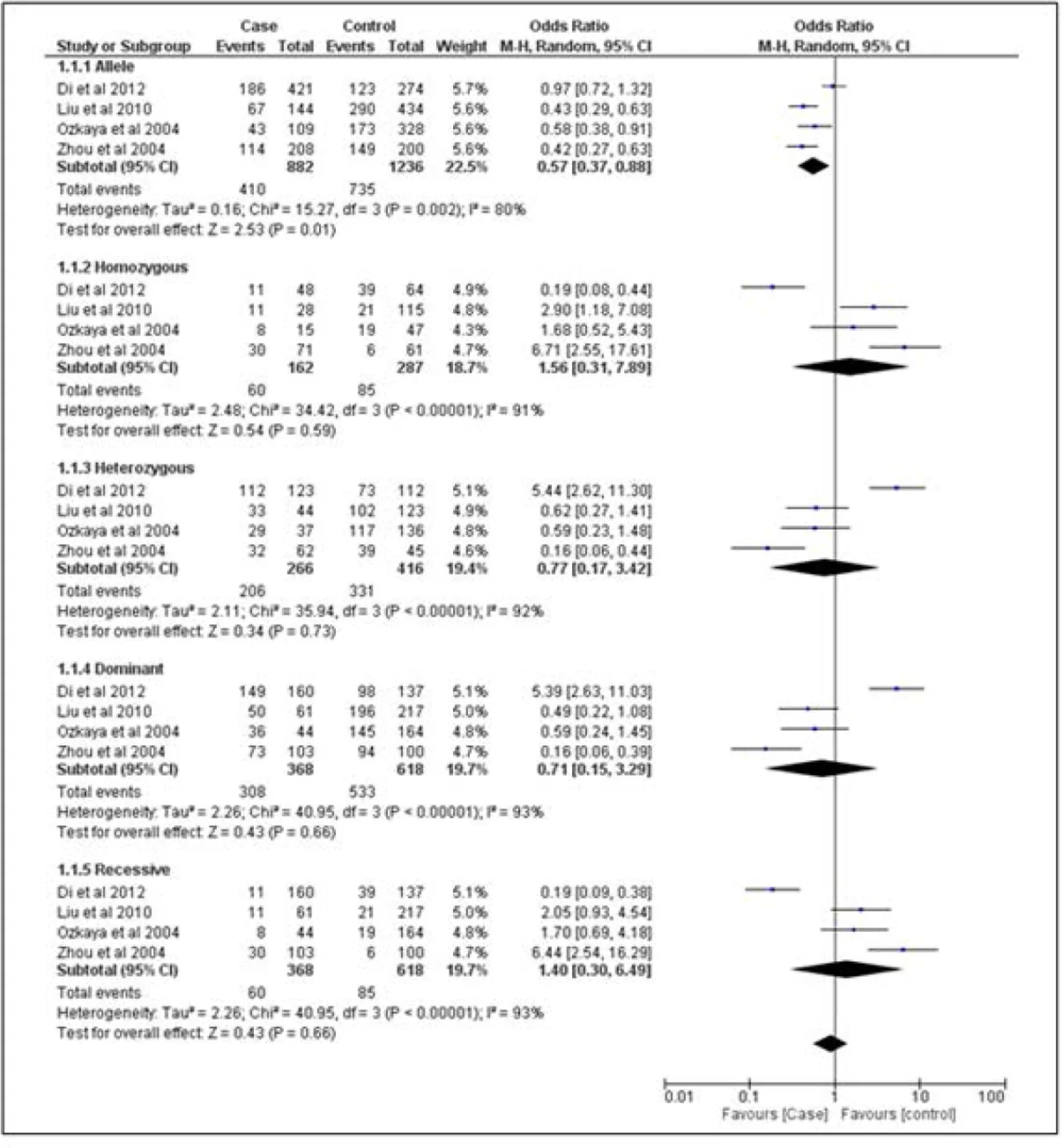
Forest plot showing an association between the ACE I/D gene polymorphism and Henoch–Schönlein purpura nephritis risk in the genetic models.

In four studies involving 368 patients with HSPN and 618 healthy controls, the polymorphism of this gene in HSPN was examined.^21,23,25,26^ Using a random-effects model, the analysis demonstrated a correlation at the allele level (P = 0.01, OR = 0.57, 95% CI [0.37, 0.88], I2=80%); however, no substantial correlation was observed in the homozygous (P = 0.59, OR = 1.56, 95% CI [0.31, 7.89], I2=91%), heterozygote (P = 0.73, OR = 0.77, 95% CI [0.17, 3.42, I2=92%]), recessive (P=0.66, OR = 1.40, 95% CI [0.30, 6.49, I2=93%]), and dominant (P=0.66, OR = 0.71, 95% CI [0.15, 3.29], I2=93%) genotypes (Figure 3).

### ACE I/D polymorphism with BD

**Figure 4.**
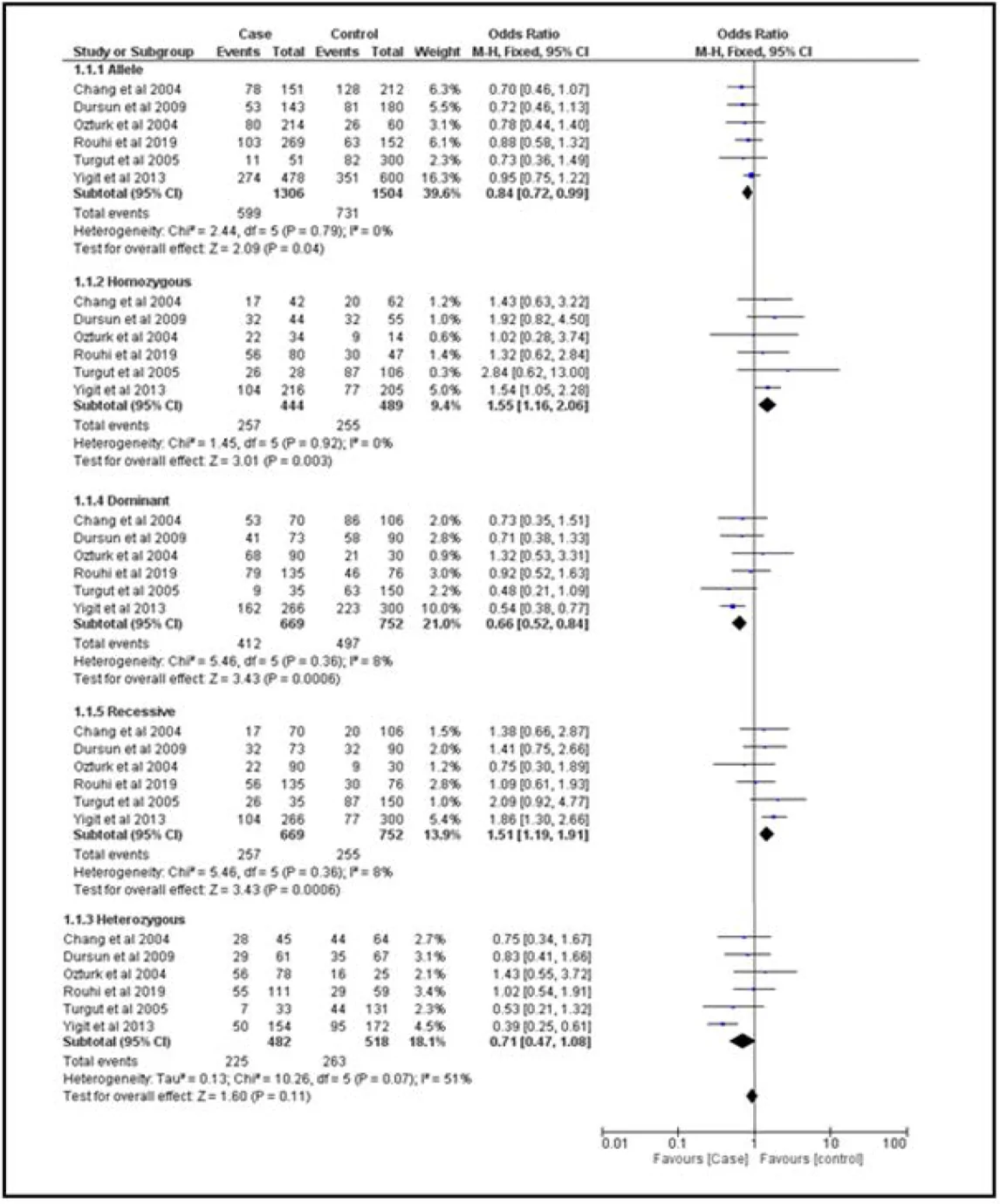
Forest plot showing an association between the ACE I/D gene polymorphism and Behçet's disease risk in the genetic models.

This polymorphism has been extensively studied in patients with BD. A meta-analysis of six studies, which included 669 BD cases and 752 healthy controls,^27–32^ revealed a substantial allelic correlation (P = 0.04, OR = 0.84, 95% CI [0.72, 0.99], I^2^ = 0%) (p = 0.0006, OR = 0.66, 95% CI [0.,52 0.84], I^2^ = 8%). homozygote (P =0.003, OR = 1.55, 95% CI [1.16,2.06, I2=0%]), and recessive (P=0.0006, OR = 1.51, 95% CI [1.19,1.91, I2=8%]) groups using the fixed effects model. Similar results were observed in the heterozygote (P = 0.002, OR = 0.65, 95% CI [0.49,0.85, I2=51%]) groups using the random-effects model, as depicted in Figure 4. Subgroup analyses of Asian and Caucasian populations revealed that the dominant model of Caucasian populations indicated a substantial correlation (P = 0.0004, OR = 0.60, 95% CI [0.46, 0.80, I2=20%]), but not in the Asian population (P = 0.4, OR = 0.84, 95% CI [0.53, 1.31, I2=0%]) (**Table 2**).

### An examination of sensitivity analysis and publication bias

**Figure 5.**
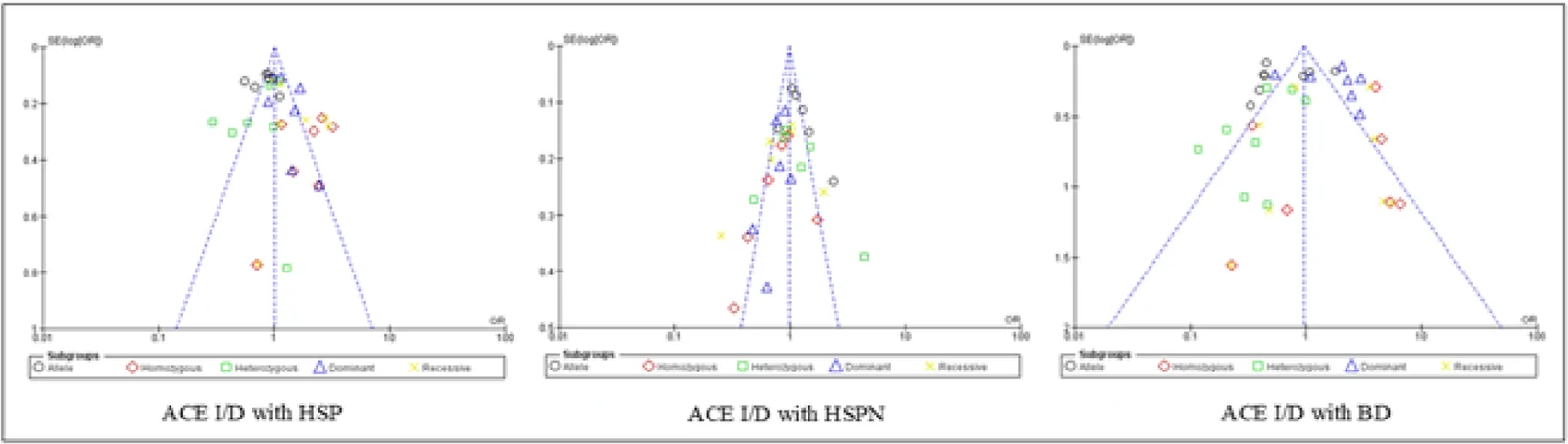
Funnel plots of publication biases on the relationships between ACE I/D polymorphism and vasculitis risk.

**Figure 6.**
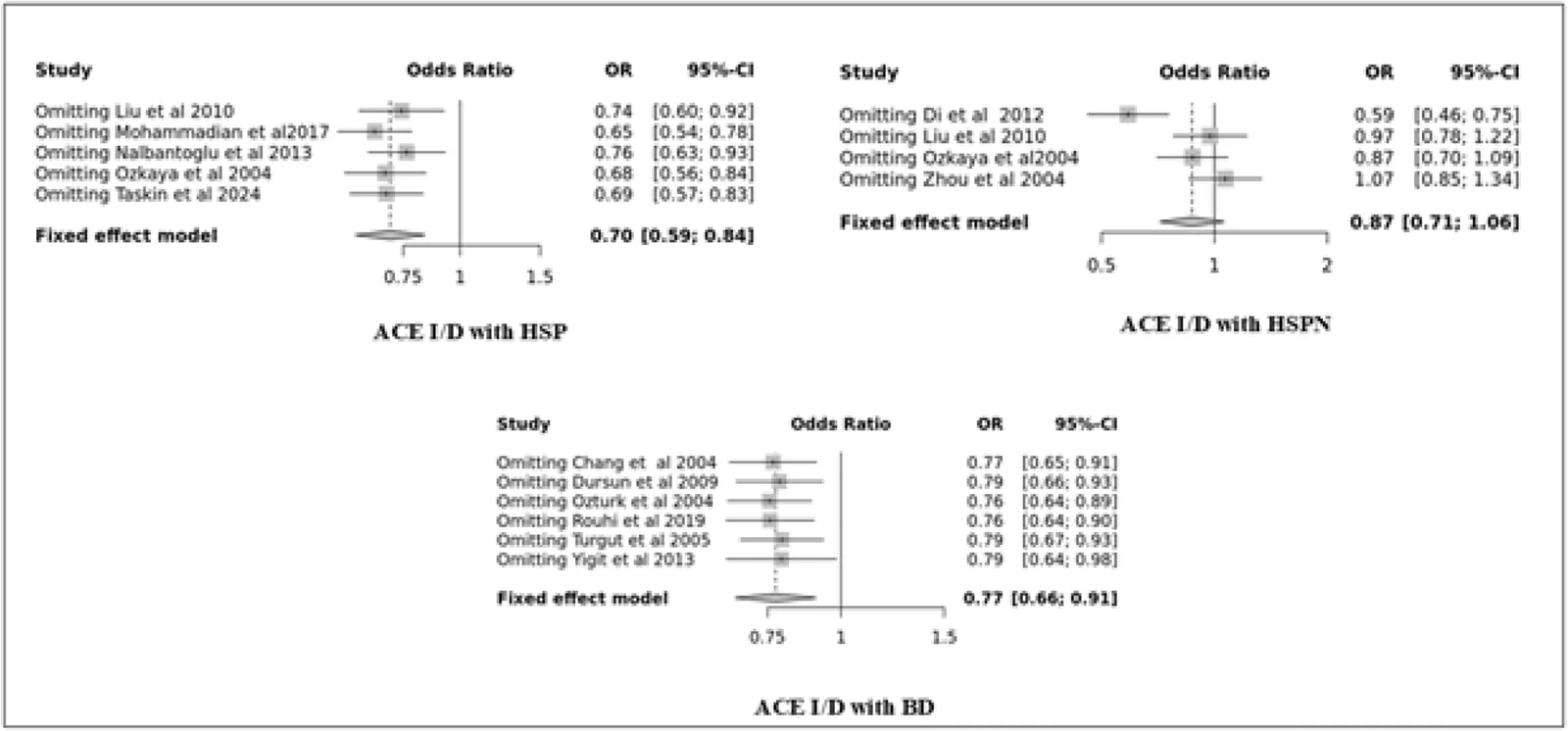
Sensitivity analysis of genetic variants of ACE I/D polymorphism and vasculitis risk.

Egger’s test and Begg’s funnel plot were applied to the included studies to examine any signs of publication bias. As supported by the results of the Begg’s test, the funnel plots for all five genetic models showed no apparent asymmetry, indicating the absence of publication bias for this polymorphism. However, some degree of asymmetry was noted in the individual studies for these polymorphisms, which was further validated using the Egger’s linear regression test. These findings were statistically robust and reliable (**Figure 5**). Additionally, a sensitivity analysis was conducted to examine the impact of individual studies on the overall pooled estimates by systematically excluding studies one at a time, including those that did not conform to the Hardy-Weinberg Equilibrium (HWE). The results indicated that the omission of any individual study did not significantly alter the overall outcomes, thereby supporting the strength and consistency of the meta-analysis findings (**Figure 6**).

### Power analysis

**Figure 7.**
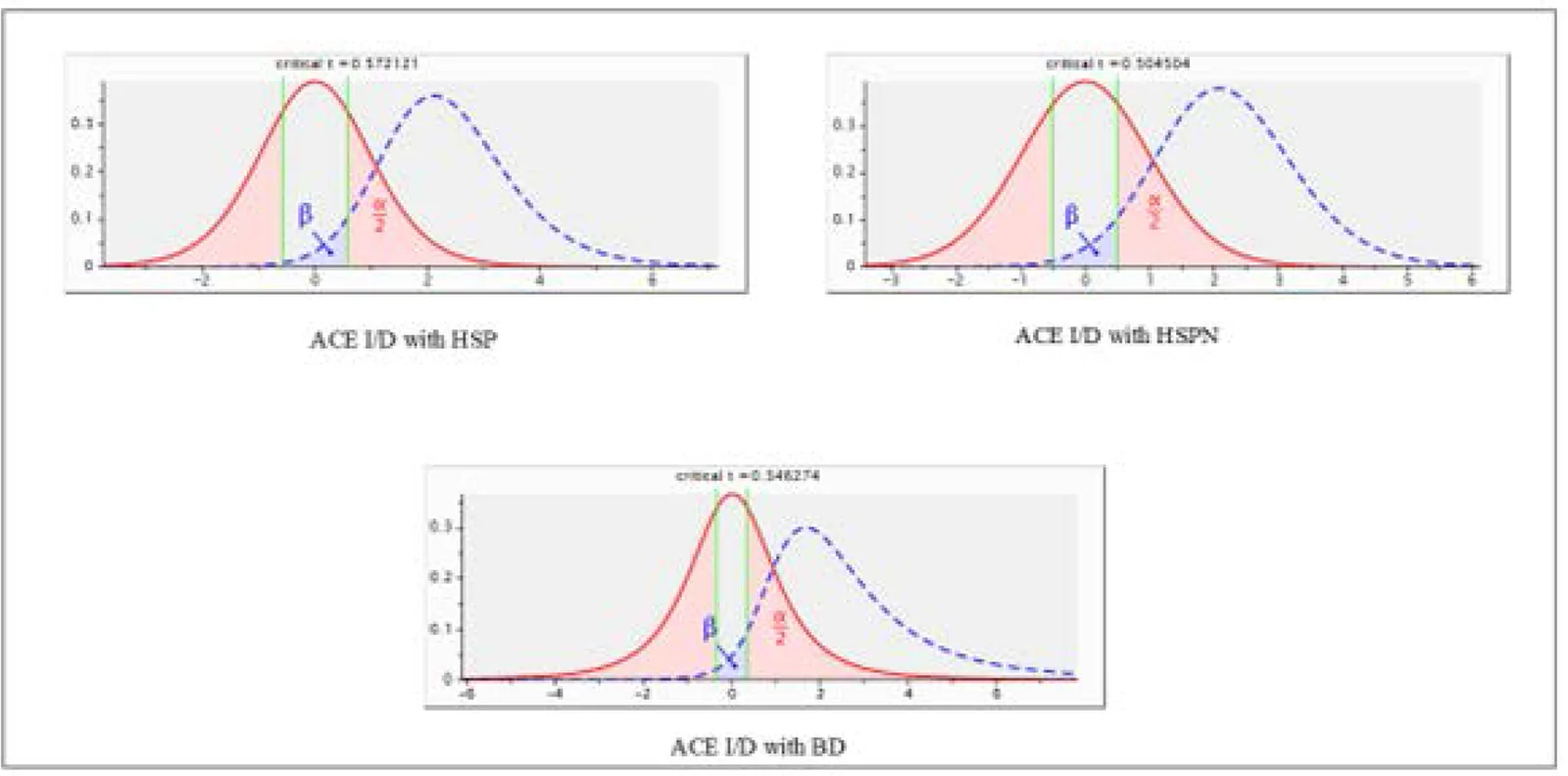
Power analysis for ACE I/D gene polymorphism and vasculitis risk.

Power analysis ensured that the included studies were adequately powered to reveal significant associations between the selected SNPs. The analysis demonstrated that the sample sizes reported in the literature were sufficient to achieve the desired level of statistical significance, with an alpha (α) error probability of 0.05, as determined using the G*Power 3.1 software. The detailed results of this analysis are presented in **Table 3**. Additionally, the power analysis graph illustrates the results of a two-tailed hypothesis test, demonstrating the likelihood of detecting the effects of a given magnitude. This is based on key parameters, including the sample size, effect size, and chosen significance level, thereby visually representing the statistical power of the study. The results suggest that the sample sizes of the included studies were sufficient to meet the required significance level, thereby enhancing the credibility and strength of our findings. This rigorous evaluation confirmed that the meta-analysis possessed adequate statistical power to identify significant associations between the investigated SNPs (**Figure 7**).

**Table 3.** Power analysis for ACE I/D gene polymorphism and vasculitis risk.

Gene	Disease	No of studies	Cases	Controls	A-error prob	Power (1-β error prob)
ACE I/D	HSP	5	499	577	0.05	0.9
ACE I/D	HSPN	4	368	618	0.05	0.9
ACE I/D	BD	6	669	752	0.05	0.9

### Gene co-expression network analysis

**Figure 8.**
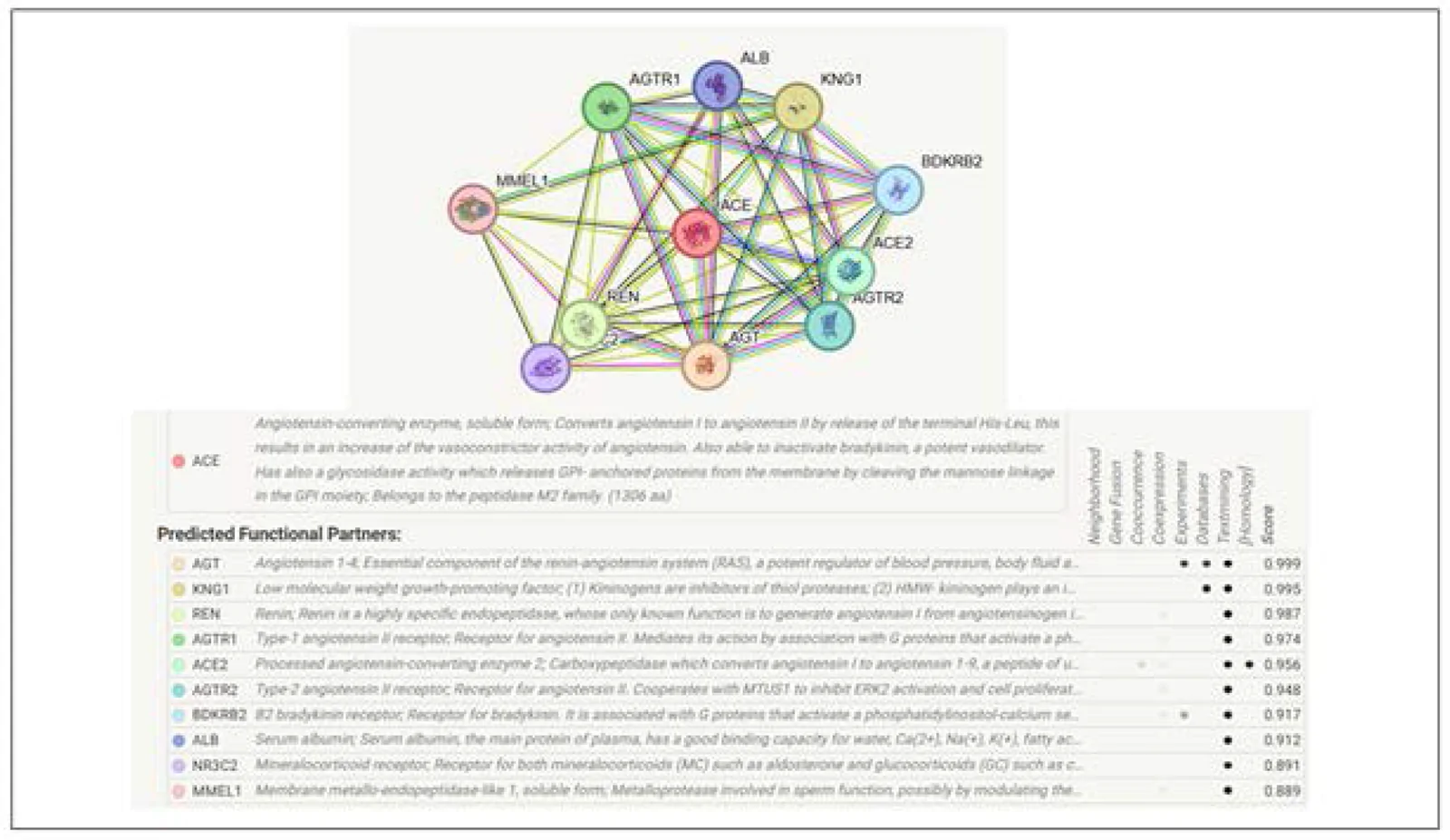
Experimentally determined direct interacting partners of the ACE I/D gene, visualised using STRING tool.

Gene–protein interaction analysis of ACE was performed using the STRING database to explore the functionally related molecules within the renin–angiotensin system. The resulting network included key interacting genes, such as AGTR1, AGTR2, REN, and KNG1, which are biologically linked to vascular regulation, endothelial function, and inflammatory signaling pathways (**Figure 8**). Although this analysis did not directly assess genetic polymorphisms, it provided a functional context supporting the potential involvement of ACE-related pathways in the pathophysiology of vasculitis.

## DISCUSSION

Previous studies on vasculitic disorders have enhanced our understanding of vascular involvement in systemic vasculitic disorders, such as microRNA expression profiles in vasculo-Behçet’s disease, highlighting the underlying vascular and inflammatory mechanisms. For example, Kaymaz et al. demonstrated that miR-195 and other microRNAs are associated with vascular manifestations in Behçet’s disease, suggesting vascular biomarkers beyond clinical features.^33^

However, a comprehensive quantitative synthesis of genetic susceptibility factors influencing vasculitis risk remains limited. The present systematic review and meta-analysis fills this gap by evaluating the association between the ACE insertion/deletion polymorphism and susceptibility to immune-mediated vasculitic disorders, including Henoch–Schönlein purpura, Henoch– Schönlein purpura nephritis, and Behçet’s disease. By integrating evidence across populations and conducting disease- and ethnicity-specific subgroup analyses, our study clarifies the inconsistent findings of previous individual genetic association studies.

The I/D polymorphism of ACE is closely related to the risk of various autoimmune disorders, including Kawasaki disease and lupus nephropathy in children.^34,35^ It has been hypothesised that this polymorphism affects the regulation of angiotensin II, potentially contributing to the pathogenesis of vasculitis. A previous meta-analysis identified the *ACE* D allele as a potential genetic risk factor associated with increased susceptibility to vasculitis; however, it did not include a subgroup analysis specifically focused on different forms of vasculitis.^17^ Recent studies have suggested a correlation between this polymorphism and susceptibility to specific types of vasculitis such as HSP, HSPN, and BD. However, conflicting findings across individual studies have limited our ability to draw definitive conclusions. Given the clinical importance of clarifying this association, we conducted a meta-analysis to thoroughly assess the association between ACE I/D polymorphisms and the risk of developing vasculitis. We included 15 eligible case-control studies that satisfied the predefined inclusion criteria of this review. Combined analysis demonstrated a significant association between this polymorphism and a heightened risk of developing vasculitis. Furthermore, subgroup analyses based on ethnicity demonstrated a robust association in Caucasian populations, suggesting a potential ethnicity-specific genetic predisposition to the disease.

Several previous studies have reported an association between the ACE I/D polymorphism and susceptibility to HSP, particularly implicating the D allele as a potential risk factor for HSP. For example, a meta-analysis by He et al. included six studies and demonstrated a significant association between the D allele and an increased risk of HSP across multiple genetic models, suggesting a possible role for heightened ACE activity and subsequent vascular inflammation in disease susceptibility.^36^ More recently, Zhang et al. analysed six studies involving 504 patients and 706 controls and similarly reported a statistically significant association between the ACE I/D polymorphism and HSP risk, with stronger effects observed in Caucasian populations compared with Asian populations.^37^ Collectively, these findings support the hypothesis that the ACE I/D polymorphism, particularly the D allele, may contribute to HSP pathogenesis through modulation of inflammatory and immunological pathways. The present meta-analysis builds on this evidence by providing an updated quantitative synthesis of available data.

The correlation between this polymorphism and the risk of HSPN has been consistently emphasised in previous meta-analyses, underscoring the genetic contribution to susceptibility to this disease. For instance, Li et al. performed a meta-analysis that included nine studies comprising 446 HSPN cases and 877 healthy controls, revealing a statistically significant correlation between this polymorphism and an increased risk of HSPN. Their findings suggest that the D allele, which is related to elevated *ACE* activity and pro-inflammatory responses, may predispose individuals to renal involvement in HSP.^38^ Similarly, Yan et al. performed a meta-analysis involving eight studies with 415 HSPN cases and 860 controls, demonstrating that carriers of the D allele had an elevated susceptibility to HSP. Furthermore, individuals with the homozygous DD genotype exhibited a substantially increased risk compared to those with genotype II, indicating a potential dose-dependent effect of the D allele on the disease progression. Subgroup analyses by Yan et al. also highlighted that this association was particularly significant among Asian and Caucasian populations.^39^ Collectively, these findings reinforce the hypothesis that this polymorphism, especially the D allele and DD genotype, may serve as a genetic biomarker for heightened susceptibility to HSPN.

The precise molecular mechanisms underlying the correlation between this polymorphism and vasculitis, such as those observed in HSP and HSPN, remain to be elucidated. This polymorphism accounts for nearly half of the inconsistencies in plasma *ACE* concentrations. Individuals with the DD genotype typically exhibit Elevated ACE levels, those with the II genotype have the lowest levels, and those with the ID genotype present intermediate levels.^40^ Elevated *ACE* levels in individuals with the DD genotype can result in vascular dysfunction, partially due to the degradation of bradykinin, a potent vasodilator. Bradykinin promotes vasodilation through endothelium-derived nitric oxide (NO) pathways, and its inactivation by *ACE* reduces NO bioavailability.^41^ Consequently, a decrease in bradykinin activity impairs NO-mediated vasodilation, which can contribute to arterial stiffness and endothelial dysfunction. These vascular alterations may foster a pro-thrombotic and pro-inflammatory environment by enhancing platelet aggregation, facilitating monocyte adhesion, promoting hemostasis, stimulating growth factor release, and releasing inflammatory cytokines. These alterations in vascular homeostasis facilitate the onset and progression of vascular processes, such as those observed in HSP and renal complications. Moreover, the increased local generation of angiotensin II in carriers of the DD genotype may further ex*ace*rbate vascular injury by increasing vascular smooth muscle cell proliferation, oxidative stress, and inflammation within the arterial walls. These effects are particularly relevant in micro-vascular beds, where immune complex deposition and vascular inflammation play central roles in the pathogenesis of HSP and HSPN.

From a disease course perspective, available evidence suggests that ACE activity is most relevant during the predisposition and early pathogenic phases of vasculitic disorders rather than during the established or relapsing phases. Elevated ACE activity associated with the D allele may lower the threshold for endothelial dysfunction, oxidative stress, and immune activation, thereby facilitating disease initiation in genetically susceptible individuals.^42^ However, current data do not support the use of circulating ACE levels as a clinical biomarker for active disease, disease recurrence, treatment resistance and therapeutic response. No validated ACE threshold has been established to guide diagnostic investigations or disease monitoring in patients with vasculitis. Therefore, ACE should be regarded primarily as a genetic susceptibility or risk-modifying factor rather than a diagnostic or prognostic biomarker, and its interpretation should be integrated with established clinical, immunological, and inflammatory parameters.

BD is a chronic, multisystem inflammatory condition chiefly characterised by recurrent genital and oral ulcers, cutaneous lesions, and ocular involvement such as uveitis.^43^ However, the disease is not confined to mucocutaneous and ocular symptoms; it can also involve blood vessels of all calibres, joints, the central nervous system, lungs, and gastrointestinal tract.^44^ Therefore, BD is considered a form of systemic vasculitis, with both arterial and venous vessels affected. Among the hallmarks of BD are vasculitis and thrombotic events, which are closely linked to endothelial cell dysfunction, a key player in the inflammatory cascade that underpins disease pathology.^45^ In light of these mechanistic pathways, few genetic association studies have assessed the potential link between this polymorphism and susceptibility to BD. The results of our meta-analysis are consistent with those of previous investigations. For instance, Manda et al. conducted a meta-analysis encompassing five eligible studies that included 534 patients diagnosed with Behçet’s disease (BD) and 676 healthy control participants and reported a statistically substantial correlation between the D allele and increased BD susceptibility. Individuals carrying the D allele had a 32% higher risk of developing BD than those carrying the I allele (OR = 1.321, P = 0.002). Moreover, those carrying the homozygous DD genotype showed a significantly elevated risk (OR = 1.573, P = 0.004), highlighting a strong correlation between genotype and disease susceptibility.^46^ Despite these promising findings, it is essential to acknowledge that the number of studies investigating the correlation between this polymorphism and BD remains limited, and the results across studies have shown a degree of heterogeneity. In conclusion, the current body of evidence, including our results, supports the hypothesis that this polymorphism, particularly the D allele and DD genotype, may contribute to increased genetic susceptibility to Behçet’s disease. Nevertheless, additional large-scale, rigorously designed studies are necessary to validate these associations and achieve a deeper understanding of the genetic factors involved in BD susceptibility.

The present meta-analysis has several limitations that should be considered when interpreting these findings. First, the number of eligible studies available for each vasculitis subtype was small. However, vasculitic disorders are rare, and genetic association studies of these conditions remain limited. In such settings, systematic reviews and meta-analyses remain valuable tools for aggregating the available evidence and improving statistical power beyond that of individual studies. ^47^ Second, the analysis was restricted to studies published in English, which may have introduced language and publication bias. Third, substantial heterogeneity was observed among the included studies, reflecting the expected clinical and methodological variability in genetic association studies of rare diseases, including differences in study design, sample size, genotyping methods, diagnostic criteria, and population characteristics. In particular, the HSPN subgroup showed high heterogeneity, likely related to variations in paediatric age distribution, definitions of renal involvement, disease severity, and biopsy practices. As HSPN represents a renal complication of HSP, complete separation between these entities may not always be absolute in retrospective studies, which may further contribute to variability between studies. Accordingly, the pooled estimates for HSPN should be interpreted cautiously and considered exploratory rather than definitive ones. In addition, subgroup analyses were limited to Asian and Caucasian populations because of data availability, which may restrict the generalisability of the findings to other ethnic groups. Furthermore, this meta-analysis focused on single-gene associations and did not account for potential confounding factors, such as environmental exposures, epigenetic influences, or gene–gene interactions. Finally, the lack of individual-level data precluded more detailed analyses of the cumulative genetic effects. Future large-scale, well-designed studies across diverse populations are needed to validate these findings and to further elucidate the genetic architecture of vasculitis.

## CONCLUSION

This meta-analysis highlights a substantial correlation between the *ACE* D allele and heightened susceptibility to vasculitis disorders, including HSP and BD, particularly in individuals of Caucasian descent and those with BD. These findings reinforce the potential contribution of genetic predisposition to the pathogenesis of vasculitis. Future investigations should prioritise large-scale, well-structured studies integrating analyses of both gene-environment and gene-gene interactions to validate these associations and deepen our understanding of the underlying biological mechanisms of these associations. Additionally, functional investigations are warranted to elucidate the biological pathways through which the ACE I/D polymorphism may influence vascular inflammation and immune dysregulation. Broadening the scope of research to include ethnically diverse populations and integrating genetic data with clinical phenotypes and molecular biomarkers will offer a more comprehensive understanding of vasculitis pathophysiology. Moreover, exploring the interaction between genetic predispositions and environmental factors will provide critical insights into the complex multifactorial origins of vasculitis.

## Data Availability

The datasets generated and/or analysed during the current systematic review and meta-analysis are available from the corresponding author upon reasonable request. All data used in this study were extracted from previously published articles and publicly available sources.
